# Comparison of the mixed approach and medial approach in laparoscopic right hemicolectomy for right colon cancer: a retrospective study

**DOI:** 10.3389/fsurg.2026.1760586

**Published:** 2026-03-20

**Authors:** Mao-Xing Liu, Fei Tan, Shun-Yu Deng, Kechen Guo, Jia-Di Xing, Pin Gao, Kai Xu, Xiang-Qian Su

**Affiliations:** 1 Key Laboratory of Carcinogenesis and Translational Research (Ministry of Education), Department of Gastrointestinal Surgery IV, Peking University Cancer Hospital & Institute, Beijing, China; 2 State Key Laboratory of Holistic Integrative Management of Gastrointestinal Cancers, Department of Gastrointestinal Surgery IV, Peking University Cancer Hospital & Institute, Beijing, China; 3 Department of Endoscopy Center and Endoscopy Research Institute, Zhongshan Hospital, Fudan University, Shanghai, China

**Keywords:** colon cancer, disease-free survival, laparoscopic surgery, overall survival, surgery approaches

## Abstract

**Purpose:**

The study aimed to determine the 3-year disease-free survival (DFS) and overall survival (OS) outcomes of the mixed approach vs. the medial approach in laparoscopic right hemicolectomy.

**Methods:**

This retrospective study analyzed clinical data from 290 patients who underwent laparoscopic right hemicolectomies at our institution. Based on the surgical approach used, the patients were categorized into a medial approach group (*n* = 144) or a mixed approach group (*n* = 146). The primary endpoint was the 3-year DFS rate and the secondary endpoint was the 3-year OS. To minimize confounding, propensity score matching was performed based on six key variables.

**Results:**

No significant difference was found in the baseline data between the two groups. Moreover, the results showed no significant difference between the medial approach and mixed approach groups in both DFS [HR = 1.799, 95% CI (0.9673, 3.347), *p* > 0.05] and OS [HR = 1.274, 95% CI (0.5521, 2.940), *p* > 0.05], regardless of tumor stage. Multivariate analyses demonstrated that tumor location and chemotherapy completion were independent prognostic factors for DFS.

**Conclusions:**

Across tumor stages, 3-year DFS and OS rates were similar between the two surgical approach groups. Thus, considering its safety and efficacy, the mixed approach may be a preferred alternative.

## Introduction

Colorectal cancer is the third most commonly diagnosed cancer and the second leading cause of cancer-related deaths worldwide, based on GLOBOCAN 2020 data (1). In China, it is the second most prevalent malignant tumor and the fourth leading cause of cancer mortality according to recent national statistics (2), with its incidence continuing to rise in recent years. For patients with both early and advanced colon cancer, surgery represents the definitive management strategy with curative potential.

The introduction of the complete mesocolic excision concept two decades ago has clearly defined the surgical boundaries for right hemicolectomy and the extent of lymph node dissection. This approach has since gained widespread clinical acceptance and application (3). The clinical efficacy of laparoscopic surgery has also been well-established (4). The medial approach, a standardized surgical technique widely adopted in clinical practice, involves initial ligation of the central vessels in accordance with the “no-touch” principle, followed by dissection of the gastrocolic ligament and mobilization of the ascending colon (5). Advances in surgical techniques have led to the emergence of numerous operative approaches (6–8). The adoption of a mixed surgical approach is primarily motivated by the limitations of any single technique in addressing complex anatomical challenges, particularly in achieving consistent identification of key vasculature and adequate exposure of critical anatomical planes. While the mixed approach initially facilitates the dissection of Toldt's space—thereby reducing operative difficulty—the delayed ligation of the tumor-feeding vessels may contravene the “no-touch” isolation principle, potentially compromising long-term oncological outcomes.

Building upon our previous findings, which established the mixed approach as a safe and advantageous alternative to the medial approach in terms of short-term surgical outcomes (9), this study was designed to evaluate its long-term oncological efficacy. Given the recognized importance of the “no-touch” isolation principle in optimizing survival, we hypothesized that the mixed approach would yield non-inferior 3-year disease-free survival (DFS) and overall survival (OS) compared to the medial approach. After a median follow-up of 3 years, we now report the comparative survival outcomes associated with each surgical technique.

## Methods

### Study design

**Figure 1:**
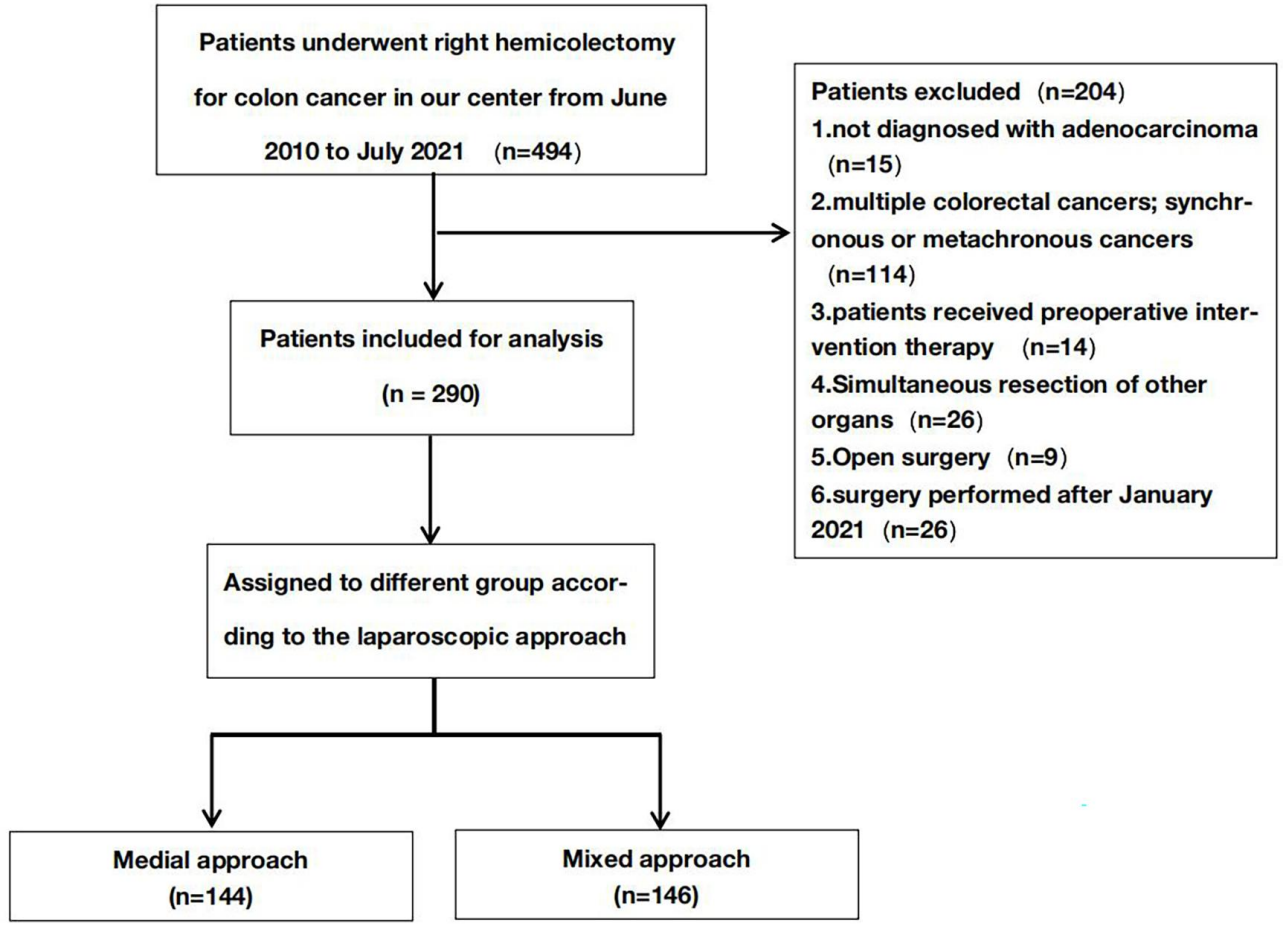
Flow chart of this study.

Adult patients with colon adenocarcinoma who underwent laparoscopic hemicolectomies at our center between June 2010 and January 2021 were retrospectively enrolled. Exclusion criteria included emergency cases or patients who had received preoperative treatments, such as endoscopic submucosal dissection, endoscopic mucosal resection, or chemotherapy. Ultimately, 290 patients were included in this study (Figure 1).

### Procedure and endpoint

A total of 144 patients underwent the medial approach, while 146 underwent the mixed approach. The surgical procedures were described in detail in our previous study. The selection of the surgical approach is determined through a comprehensive evaluation of clinical factors, including the patient's specific anatomical characteristics, lesion location, and surgeon experience, which aligns with standard clinical decision-making processes. The primary endpoint was 3-year DFS, defined as the interval from the date of surgery to the date of tumor progression (local recurrence, metastasis, or death). The secondary endpoint was 3-year OS, defined as the interval from the date of surgery to the date of death from any cause.

### Follow-up

The scheduled follow-up period was at least 36 months. However, 29 patients were followed for less than 3 years, including three who experienced recurrence or metastasis within this period. The censoring rate was 10%. Censored cases were not categorized as recurrence, metastasis, or death and were included in the final survival analysis and shown in the final images. All the patients adhered to the follow-up protocol, which involved assessments every 3 months for the first 2 years and every 6 months for the subsequent 3 years. The follow-up protocol included the following: (1) a physical examination; (2) routine blood tests and serum tumor markers, including carcinoembryonic antigen (CEA), carbohydrate antigen 19-9 (CA19-9), and carbohydrate antigen 125 (CA125); (3) imaging, such as abdominopelvic and chest computed tomography scans; and (4) annual endoscopy. Magnetic resonance imaging, positron emission tomography, or laparoscopic exploration was performed when recurrence or metastasis was suspected. Adjuvant oxaliplatin-based chemotherapy was administered to patients with pathological stage II or higher disease. The final follow-up date was 6 January 2024, with a median follow-up duration of 3.42 years.

### Statistical analysis

The statistical analyses were performed using SPSS version 22.0, R version 4.4.2 (R Foundation for Statistical Computing), and GraphPad Prism 10. Continuous variables are expressed as mean ± standard deviation. Differences between groups were assessed using Student's *t*-test or the Mann–Whitney *U*-test, as appropriate. Categorical variables are presented as frequencies and were compared using the chi-square test or Fisher's exact test. The 3-year DFS rate was calculated using the Kaplan–Meier method. Univariate and multivariate analyses were performed using Cox proportional hazards regression models. In the subgroup analyses, DFS stratified by pathological stage (I, II, or III) was compared using the log-rank test. A *P*-value <0.05 was considered statistically significant. To further mitigate selection bias, propensity score matching was conducted.

## Results

### Preoperative evaluation

Of 494 patients with colon cancer, 290 cases were selected, namely 144 cases in the medial approach group and 146 cases in the mixed approach group. The average age of the former was 59.65 years and of the latter was 58.12 years. Men accounted for 64% (*n* = 92) of the medial approach patients and 55% (*n* = 80) of the mixed approach patients. There were 45 patients with hypertension, 23 patients with diabetes, and 129 patients with American Society of Anesthesiologists (ASA) grade II or above in the medial approach group. Preoperative hemoglobin concentration values and preoperative tumor markers are listed in Table 1. The results indicated no significant differences between the two groups, confirming that the baseline characteristics were comparable.

**Table 1:** Baseline clinical characteristics of the included patients.

Variable	Medial approach (*n* = 144)	Mixed approach (*n* = 146)	*P*-value
Age (years)	59.65 (SD12.81)	58.12 (SD12.96)	0.313
Gender (male)	92 (63.9%)	80 (54.8%)	0.115
Body mass index (kg/m^2^)	23.99 (SD3.29)	23.16 (SD3.23)	
Hypertension	45 (31.2%)	41 (28.1%)	0.555
Diabetes	23 (16%)	15 (10.3%)	0.150
ASA			
I	15 (10.4%)	20 (13.7%)	0.441
II	120 (83.3%)	113 (77.4%)	
III	9 (6.2%)	13 (8.9%)	
Preoperative hemoglobin concentration (g/L)	108.83 (SD22.65)	110.72 (SD22.80)	0.477
CEA (ng/mL)	15.97 (SD53.88)	8.30 (SD4.30)	0.128
CA199 (U/mL)	36.77 (SD101.85)	47.95 (SD150.46)	0.501
CA724 (U/mL)	16.01 (SD71.66)	13.24 (SD50.30)	0.727
CA242 (U/mL)	20.78 (SD36.86)	25.94 (SD59.64)	0.444

### Pathological characteristics

All the surgical specimens underwent comprehensive pathological evaluations. In the medial approach group, the mean maximum and minimum tumor diameters were 5.56 and 4.1 cm, respectively. Tumor locations included the ileocecal region (*n* = 36), ascending colon (*n* = 60), and hepatic flexure/transverse colon (*n* = 48). In the mixed approach group, the corresponding mean diameters were 5.43 and 4.11 cm, with tumors located in the ileocecal region (*n* = 31), ascending colon (*n* = 79), and hepatic flexure colon (*n* = 36). Pathological assessment yielded an average of 25.68 harvested lymph nodes. All patients were classified as American Joint Committee on Cancer (AJCC) stages I–III, with a comparable stage distribution between the two cohorts. Detailed TNM staging and adjuvant chemotherapy data are presented in Table 2.

**Table 2:** Postoperative pathological data of the included patients.

Variable	Medial approach (*n* = 144)	Mixed approach (*n* = 146)	*P*-value
Maximum tumor diameter (cm)	5.56 (SD2.38)	5.43 (SD2.22)	0.643
Minimum tumor diameter (cm)	4.1 (SD1.7)	4.1 (SD1.75)	0.978
Location, *n* (%)			
Ileocecal region	36 (25)	31 (21.2)	0.097
Ascending colon	60 (41.7)	79 (54.1)	
Hepatic flexure	48 (33.3)	36 (24.7)	
Lymph node harvest	25.68 (SD11.53)	25.45 (SD9.718)	0.851
Metastatic lymph nodes	1.38 (SD2.536)	0.95 (SD2.010)	0.111
pTNM, *n* (%)			
1	18 (12.5)	14 (9.6)	0.172
2	71 (49.3)	88 (60.3)	
3	55 (38.2)	44 (30.1)	
T stage, *n* (%)			
1	7 (4.9)	2 (1.4)	0.172
2	13 (9)	15 (10.3)	
3	91 (63.2)	103 (70.5)	
4	33 (22.9)	26 (17.8)	
N stage, *n* (%)			
0	89 (61.8)	102 (69.9)	0.283
1	34 (23.6)	30 (20.5)	
2	21 (14.6)	14 (9.6)	
Received adjuvant chemotherapy, *n* (%)			
II	47 (66.20)	64 (72.73)	ns
III	55 (100)	44 (100)	
Regular chemotherapy, *n* (%)			
II	42 (89.36)	56 (87.50)	ns
III	43 (78.18)	34 (77.27)	

### Oncological outcomes

**Figure 2:**
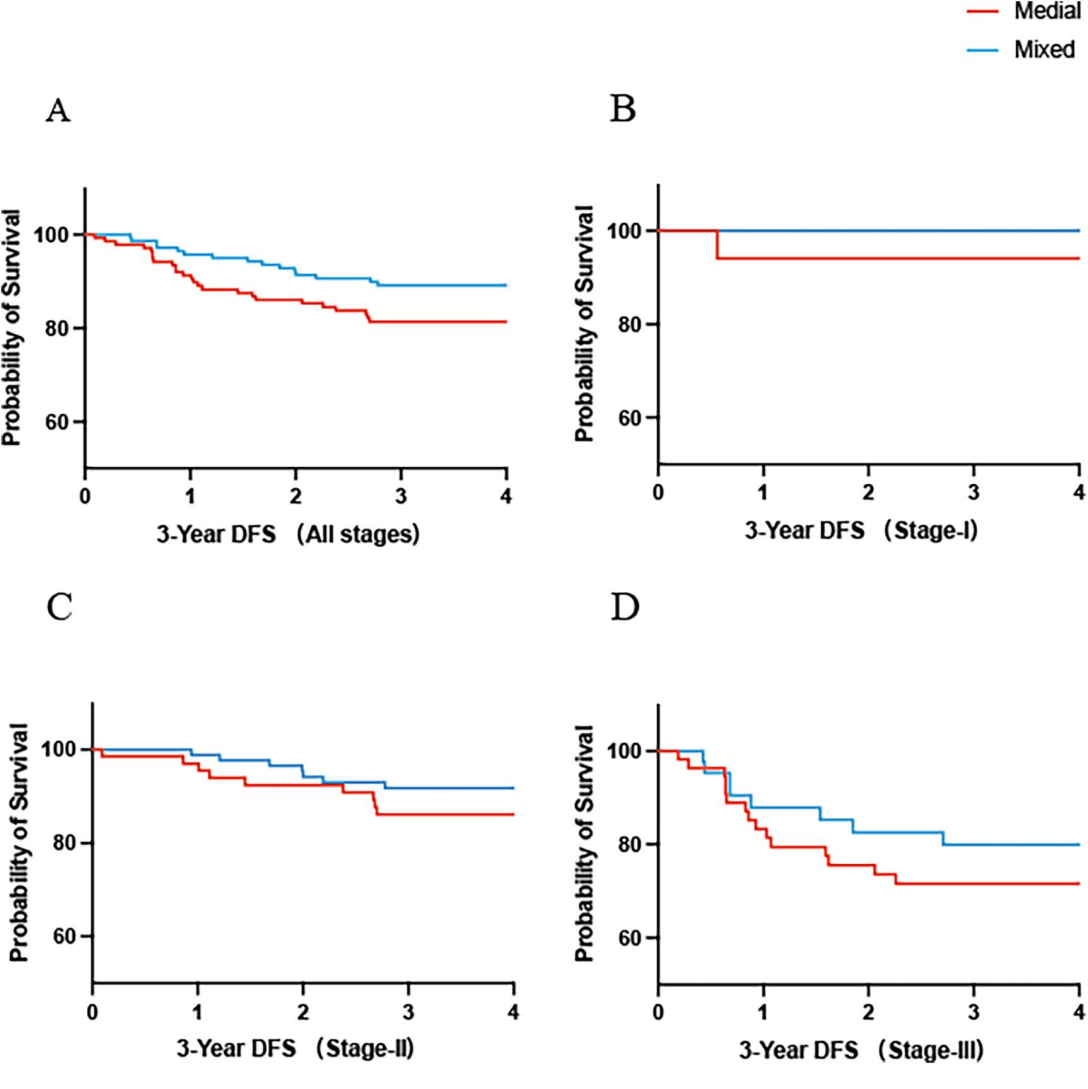
The 3-year DFS rate in the medial group and mixed group of all patients **(A)**. The 3-year DFS rate in the medial group and mixed group stratified by pathologic staging I **(B)**, II **(C)**, III **(D)**.

**Figure 3:**
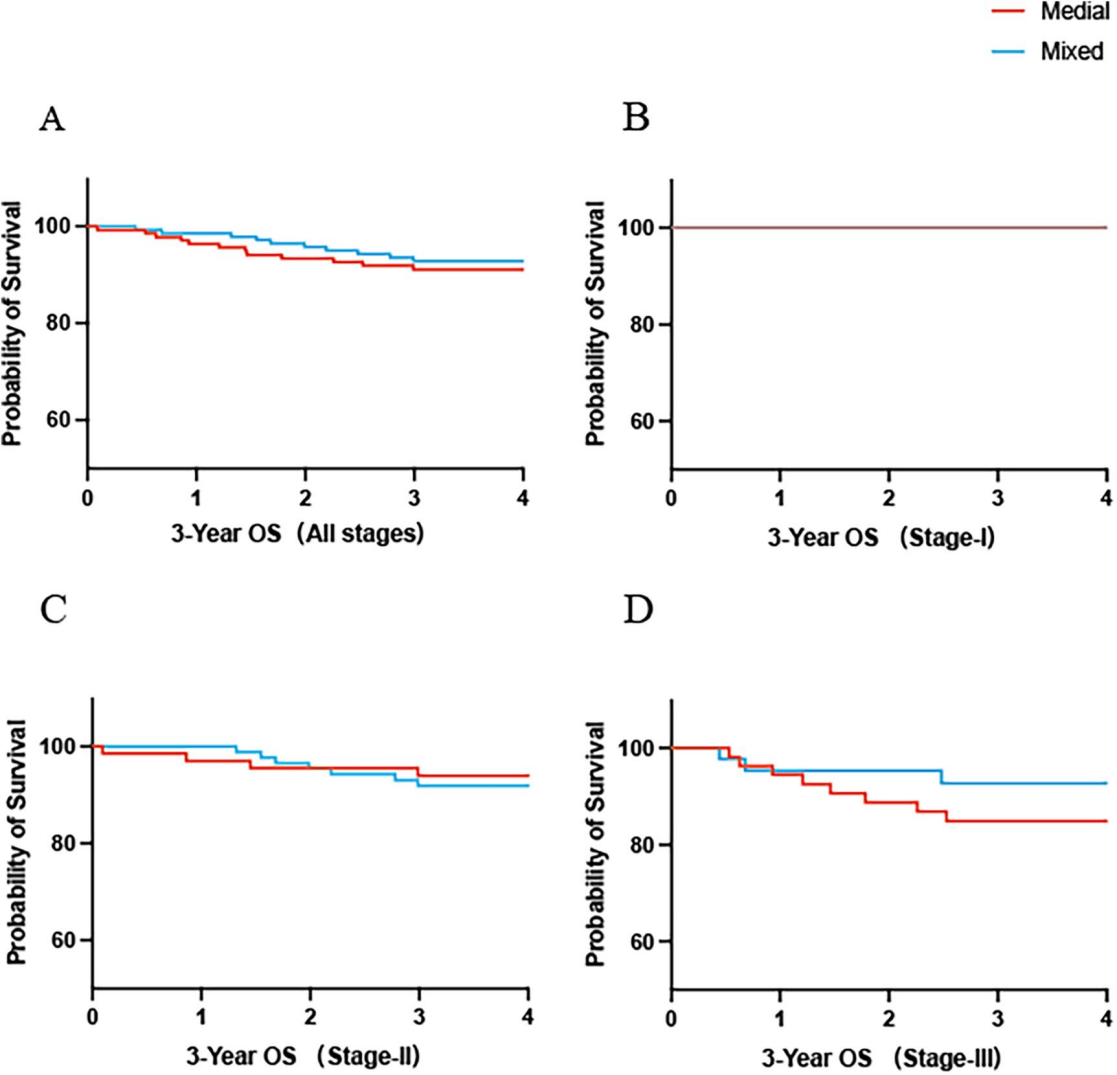
The 3-year OS rate in the medial group and mixed group of all patients **(A)**. The 3-year OS rate in the medial group and mixed group stratified by pathologic staging I **(B)**, II **(C)**, III **(D)**.

The 3-year DFS rate was similar between the medial and mixed approach groups [HR = 1.80, 95% CI (0.97, 3.35), *p* > 0.05] (Figure 2A). Subgroup analyses according to pathological stage also showed no statistical differences between the two groups for stage I [HR = 5.84, 95% CI (0.11, 304.9), *p* > 0.05], stage II [HR = 1.76, 95% CI (0.65, 4.75), *p* > 0.05], or stage III [HR = 1.47, 95% CI (0.64, 3.35), *p* > 0.05] (Figures 2B–D). A similar trend was observed for OS [HR = 1.27, 95% CI (0.55, 2.94), *p* > 0.05]. Furthermore, no significant differences were found in the stage-specific OS analyses for stage I, stage II [HR = 0.75, 95% CI (0.23, 2.49), *p* > 0.05], or stage III [HR = 2.00, 95% CI (0.61, 6.57), *p* > 0.05] (Figure 3).

### Risk factors for survival

Univariate and multivariate analyses of risk factors for survival are presented in Table 3. Univariate analysis indicated that the N stage as a whole significantly influenced survival outcomes (*p* = 0.0025). However, in the multivariate Cox regression analysis—which adjusted for age, gender, BMI, tumor location, T stage, N stage, and completion of adjuvant chemotherapy—neither specific N substages [N1: HR = 1.42, 95% CI [0.63, 3.19], *p* > 0.05; N2: HR = 0.42, 95% CI [0.11, 1.56], *p* > 0.05] nor the number of positive lymph nodes demonstrated independent prognostic significance.

**Table 3:** Univariate and multivariate Cox regression analyses of the risk factors for survival.

Variable		Univariate analysis			Multivariate analysis		
		HR	95% CI	*P*	HR	95% CI	*P*-value
Age		1.0021	0.9787–1.0261	0.8623	0.9951	0.9705–1.0204	0.7026
BMI		1.0198	0.9279–1.1207	0.6843	1.0312	0.9229–1.1521	0.5877
Maximum diameter		1.0541	0.9218–1.2054	0.4414			
Female		1.5571	0.8376–2.8945	0.1615	0.9491	0.4883–1.8448	0.8775
Surgical approach		1.2264	0.6578–2.2865	0.5208			
Number of positive lymph nodes		0.9002	0.7547–1.0736	0.2420			
N stage				**0** **.** **0025**			
N1		0.9803	0.4626–2.0776	0.9587	1.4190	0.6318–3.1873	0.3966
N2		0.5168	0.1571–1.7002	0.2773	0.4184	0.1125–1.5555	0.1934
Chemotherapy completion		0.4485	0.2396–0.8397	**0** **.** **0122**	0.4032	0.1950–0.8335	**0** **.** **0142**
T stage	T1						
	T2	0.9111	0.1767–4.6964	0.9114	1.9959	0.3716–10.7214	0.4204
	T3	0.6108	0.1449–2.5737	0.5017	2.0378	0.4595–9.0376	0.3489
	T4	0.5421	0.1126–2.6101	0.4451	2.7438	0.4977–15.1281	0.2465
Tumor location (ileocecal region)				**0** **.** **0000**			
Ascending colon		0.0894	0.0392–0.2038	**0** **.** **0000**	0.0766	0.0328–0.1785	**0** **.** **0000**
Hepatic flexure		0.0623	0.0190–0.2043	**0** **.** **0000**	0.0527	0.0157–0.1769	**0** **.** **0000**

Bold values indicate *P* < 0.05, suggesting statistical significance.

Primary tumor location and completion of adjuvant chemotherapy were identified as independent factors affecting survival. Compared with ileocecal tumors, the risk of recurrence or death was significantly lower for tumors in the ascending colon [HR = 0.077, 95% CI (0.033, 0.179), *p* < 0.001] and the hepatic flexure [HR = 0.053, 95% CI (0.016, 0.177), *p* < 0.001]. Completion of adjuvant chemotherapy reduced the risk of disease recurrence or death by approximately 60% [HR = 0.403, 95% CI (0.195, 0.834), *p* = 0.014].

### Propensity score matching

To control for potential confounding factors, propensity score matching was performed in a 1:1 ratio based on the following six variables: hypertension, diabetes, T stage, N stage, gender, and BMI. After matching, 123 patients were included in each of the medial approach and mixed approach groups for final analysis. We analyzed the baseline pathological characteristics (Supplementary Table S1) and survival outcomes (Supplementary Figure S1) in the matched cohort and subsequently conducted univariate and multivariate Cox regression analyses (Supplementary Table S2). The results demonstrated that the principal findings from the matched analysis remained consistent with those from the preliminary full-cohort analysis.

## Discussion

This retrospective study found no statistically significant differences in 3-year DFS or OS between the mixed and medial approach groups. The multivariate analysis further demonstrated that tumor location was an independent predictor of improved survival outcomes for patients with colon cancer following surgery.

Advancements in surgical techniques have substantially improved curative rates (10–12). The medial approach was the standard right hemicolectomy technique decades ago, following the “no-touch” principle (7). However, in actual surgical practice, several technical challenges exist, including obesity and vascular anatomical variations, which may increase procedural complexity. As a standard surgical approach in our center, the mixed technique has been shown to reduce operative time, intraoperative bleeding, and postoperative drainage volume, without prolonging hospitalization or increasing postoperative complication rates, consistent with our previous report (9). However, as this approach does not prioritize the early ligation of central vessels, concerns have been raised that intraoperative manipulation and compression of the tumor could potentially lead to hematogenous tumor dissemination. Our previous studies have clarified the advantages of this approach in terms of surgical duration and safety (9). After a 3-year follow-up period, the mixed approach was not associated with an increased risk of recurrence or metastasis.

To evaluate the oncological efficacy of different surgical approaches in laparoscopic right hemicolectomy, this study selected disease-free survival as the primary endpoint based on the following considerations. First, DFS directly reflects the fundamental goal of curative surgery—complete tumor removal and prevention of recurrence—and can more sensitively and quickly capture potential differences in oncological outcomes attributable to variations in surgical technique. Second, consistent with previous reports (11–13), DFS is a well-validated and widely accepted endpoint in international prospective large-scale studies, and its improvement has been consistently shown to predict long-term overall survival benefit, particularly in trials comparing treatment strategies aimed at reducing recurrence risk. Furthermore, compared to overall survival, DFS is less confounded by non-cancer-related mortality, allowing a more focused assessment of the antitumor effect of the intervention itself. Therefore, adopting DFS as the primary endpoint enables a more efficient and precise evaluation of potential differences in tumor control between surgical approaches, providing clinically actionable evidence to guide practice. Our DFS outcomes were comparable to published rates, such as the 81.7% reported in another right hemicolectomy study (12). While some previous studies have suggested that different surgical approaches may influence patient survival outcomes (14), our initial analysis indicated that the medial approach was associated with a trend toward poorer survival compared to the mixed approach, though this difference did not reach statistical significance [HR = 1.799, 95% CI (0.967, 3.347), *p* > 0.05]. Given the well-established prognostic impact of tumor stage, we further conducted stage-stratified survival analyses to account for potential imbalances in stage distribution between the two groups. These analyses further demonstrated that for patients with different tumor stages, including those with locally advanced disease (stages II and III), both surgical approaches yielded comparable survival outcomes, supporting the oncological safety of both techniques. In addition, to mitigate selection bias and control for other confounding factors, we performed propensity score matching based on key clinicopathological variables and repeated the survival analysis in the matched cohort. These complementary analytical approaches—stage stratification and propensity score matching—enhanced the robustness and validity of our findings, confirming that after adjusting for tumor stage and other prognostic covariates, the surgical approach itself did not independently affect survival outcomes.

Based on the Cox regression analysis of the cohort (*n* = 290, events = 40), the surgical approach was not significantly associated with disease-free survival (HR = 1.23, 95% CI 0.66–2.29, *p* = 0.52). The result indicated that the choice of surgical approach did not independently influence DFS outcomes in this study population.

Furthermore, while the univariate analysis indicated that lymph node metastasis (N stage) was associated with survival outcomes—consistent with its established role in the AJCC staging system, 8th edition, where any nodal involvement is classified as at least stage III disease—this association did not retain statistical significance in the multivariate model after adjusting for other prognostic variables. In contrast, completion of adjuvant chemotherapy emerged as a significant independent factor influencing survival in the multivariate analysis, underscoring its critical role in determining patient prognosis beyond nodal status alone. Clinically, nodal status directly informs adjuvant chemotherapy decisions. In clinical practice, patients with lymph node metastasis are classified as at least stage III and, barring contraindications, are routinely recommended to receive adjuvant chemotherapy. Substantial evidence (15–17) confirms that completing adjuvant chemotherapy improves patient prognosis. Consequently, nodal stage and chemotherapy completion are intrinsically linked in the clinical pathway. In the multivariate regression, it is therefore understandable that the prognostic signal initially carried by nodal stage may be absorbed by chemotherapy completion—a more proximal and directly actionable treatment factor—leading to the loss of independent statistical significance for nodal stage in the adjusted model.

Increased risk due to a tumor located in the ileocecal region has not been previously reported. According to our research results, when tumors were located in the ileocecal region rather than the hepatic flexure, they were associated with a 3.77-fold increased risk. In order to investigate the possible reason, we conducted a subgroup analysis. A total of 67 patients had tumors located in the ileocecal region, among which 42 patients (62.7%) did not have lymph node metastasis, while the proportion in the group who had tumors located in the hepatic flexure was 67.9% (57/84). Among them, 7 (10.4%) patients in the former group were classified as pTNM stage I, while 11 (13.1%) patients in the latter group were pTNM stage I. Even though the ileocecal tumor group had a lower proportion of patients without lymph node metastasis or in pathological stage I, they still exhibited a lower DFS rate compared to the group with tumors located at the hepatic flexure. Given the known differences in clinical manifestations and survival outcomes between left- and right-sided colon cancer (18), we hypothesize that tumors located in the ileocecal region may be a potential risk factor for survival. We acknowledge that the observed association between ileocecal tumor location and inferior DFS, while notable, remains speculative in its biological mechanism. Therefore, this finding should be interpreted as a hypothesis-generating observation rather than a definitive conclusion. Further validation in larger, multicenter cohorts is warranted to confirm its prognostic significance and elucidate the underlying biological rationale.

Several limitations of this study should be considered. First, as this was a retrospective study in a single center, more randomized controlled trials (RCTs) are needed in the future to validate this study’s findings. Second, only 290 cases were included in this study and the sample size was small. Finally, the 3-year follow-up was relatively short, and long-term follow-up is necessary in the future.

## Conclusions

In conclusion, this study demonstrates that the mixed approach is oncologically non-inferior to the medial approach, with comparable 3-year survival outcomes across tumor stages.

## Data Availability

The raw data supporting the conclusions of this article will be made available by the authors, without undue reservation.

## Supplementary material

Supplementary Figure S1

The 3-year OS rate in the Medial group and Mixed group of all patients (A); The 3-year OS rate in the in the Medial group and Mixed group stratified by pathologic staging I (C), II (E), III (G); The 3-year DFS rate in the Medial group and Mixed group of all patients(B); The 3-year DFS rate in the in the Medial group and Mixed group stratified by pathologic staging I (D), II (F), III (H).
