# Supplementary figures and images for: Comparison of the mixed approach and medial approach in laparoscopic right hemicolectomy for right colon cancer: a retrospective study
Source: Front Surg. 2026 Mar 20;13:1760586. doi: 10.3389/fsurg.2026.1760586 (PMC13047092; doi:10.3389/fsurg.2026.1760586)

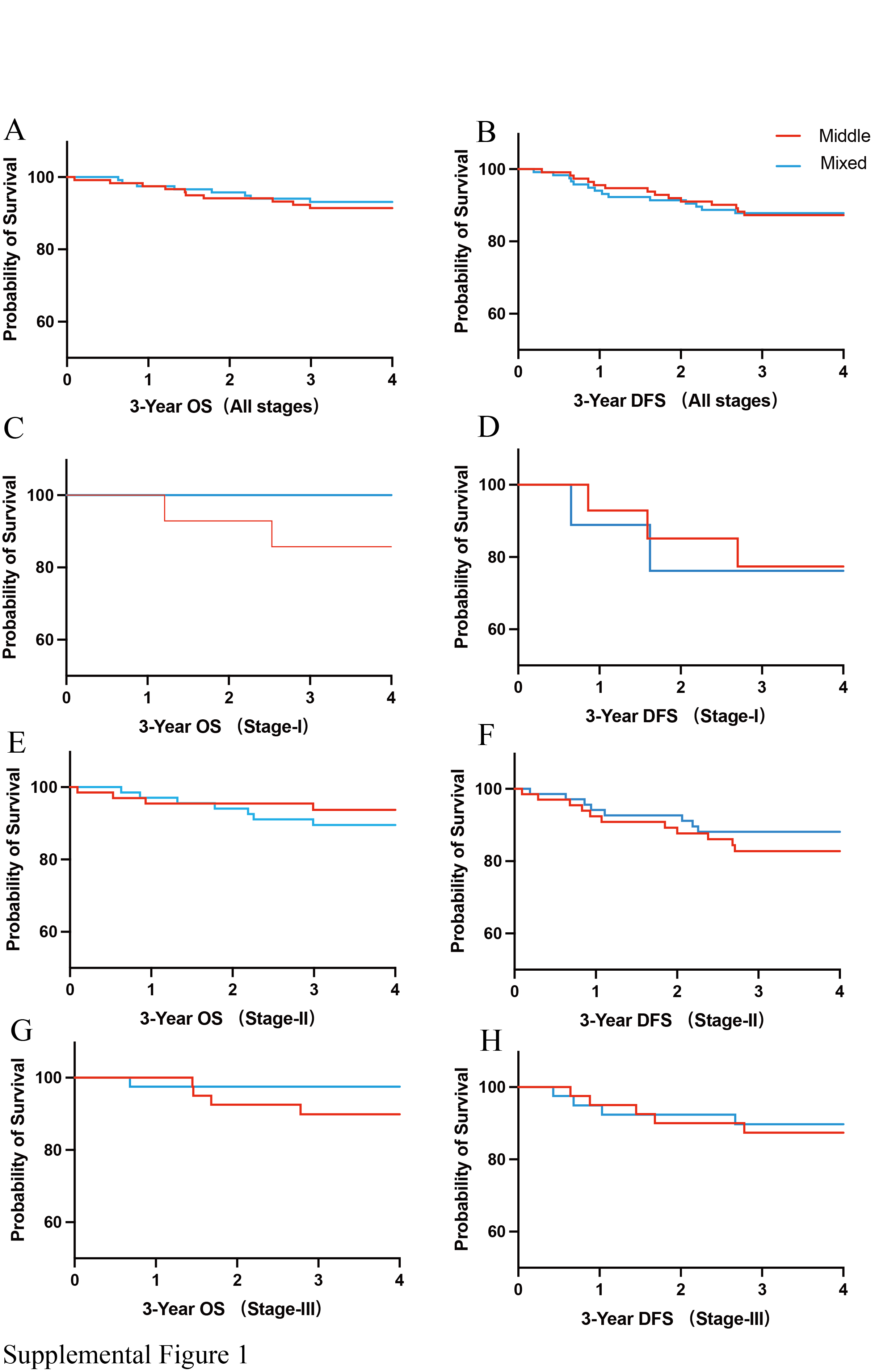

Supplement: Supplementary file 2 [file Image1.png]
