# Supplementary material for: Comparison of the mixed approach and medial approach in laparoscopic right hemicolectomy for right colon cancer: a retrospective study
Source: Front Surg. 2026 Mar 20;13:1760586. doi: 10.3389/fsurg.2026.1760586 (PMC13047092; doi:10.3389/fsurg.2026.1760586)
Supplement: Supplementary file 3 [file Table2.docx]

Supplemental Table 2. Univariate and multivariate Cox regression analyses of risk factors for survival after PSM

| Variable |  | Univariate analysis | | | Multivariate analysis | | |
| --- | --- | --- | --- | --- | --- | --- | --- |
|  |  | HR | 95%CI | P-value | HR | 95%CI | P-value |
| Age |  | 0.9968 | 0.9711 - 1.0233 | 0.8123 | 0.9857 | 0.9585 - 1.0137 | 0.3145 |
| BMI |  | 1.0191 | 0.9175 - 1.1320 | 0.7234 | 1.0389 | 0.9158 - 1.1785 | 0.5531 |
| Diameter_max |  | 1.0709 | 0.9284 - 1.2354 | 0.3470 |  |  |  |
| Female |  | 1.7258 | 0.8718 - 3.4165 | 0.1173 | 1.0061 | 0.4849 - 2.0879 | 0.9869 |
| Surgery approch |  | 1.3460 | 0.6748 - 2.6846 | 0.3989 |  |  |  |
| Positive_Lymph Node number |  | 0.9240 | 0.7682 - 1.1113 | 0.4012 |  |  |  |
| N stage | N0 |  |  |  |  |  |  |
|  | N1 | 0.7918 | 0.3237 - 1.9372 | 0.6091 | 1.0290 | 0.3945 - 2.6841 | 0.9534 |
|  | N2 | 0.6101 | 0.1837 - 2.0262 | 0.4197 | 0.4674 | 0.1241 - 1.7598 | 0.2608 |
| Chemotherapy completion |  | 0.4564 | 0.2288 - 0.9104 | 0.0260 | 0.4203 | 0.1897 - 0.9316 | **0.0328** |
| Tstage | T1 |  |  |  |  |  |  |
|  | T2 | 0.3031 | 0.0506 - 1.8163 | 0.1913 | 0.7770 | 0.1209 - 4.9951 | 0.7904 |
|  | T3 | 0.2965 | 0.0696 - 1.2630 | 0.1001 | 1.2082 | 0.2628 - 5.5539 | 0.8080 |
|  | T4 | 0.2672 | 0.0538 - 1.3261 | 0.1064 | 1.5939 | 0.2693 - 9.4342 | 0.6073 |
| Tumor_location（ileocecal） |  |  |  |  |  |  |  |
| ascending colon |  | 0.0914 | 0.0375 - 0.2224 | 0e+00 | 0.0831 | 0.0333 - 0.2072 | **0.0000** |
| hepatic flexure |  | 0.0244 | 0.0033 - 0.1798 | 3e-04 | 0.0212 | 0.0028 - 0.1596 | **0.0002** |
